# Long noncoding RNA endogenous bornavirus-like nucleoprotein acts as an oncogene by regulating microRNA-655-3p expression in T-cell acute lymphoblastic leukemia

**DOI:** 10.1080/21655979.2022.2044249

**Published:** 2022-02-27

**Authors:** Jinhua Yang, Yingying Yang

**Affiliations:** Department of Hematology, Wuhan Fourth Hospital, Puai Hospital, Tongji Medical College, Huazhong University of Science and Technology, Wuhan, Hubei, China

**Keywords:** lncRNA EBLN3P, T-ALL, miR-655-3p, tumor proliferation, tumor invasion, metastasis

## Abstract

Acute lymphocytic leukemia (ALL) is the most common malignant tumor in children with T-cell ALL (T-ALL), accounting for approximately 15% of all cases. Long noncoding RNAs (lncRNAs) are involved in the pathogenesis and progression of T-ALL. The present study aimed to explore the role and mechanism of action of lncRNA EBLN3P in T-ALL. We used quantitative reverse transcription-PCR (qRT-PCR) to determine the expression of lncRNA endogenous bornavirus-like nucleoprotein (EBLN3P), microRNA (miR)-655-3p, and the transcription level of matrix metalloproteinase-9 (MMP-9), and Western blot assay to quantify the protein expression level of cleaved-caspase3, caspase3, proliferating cell nuclear antigen (PCNA), and MMP-9. The potential binding sites between lncRNA EBLN3P and miR-655-3p were predicted using StarBase, and the interaction was further verified by dual-luciferase reporter assay and RNA pull-down assay. The proliferation ability of Jurkat cells was detected using MTT (3-(4,5-dimethylthiazol-2-yl)-2,5-diphenyltetrazolium bromide) assay, and their invasion and migration ability using transwell assay. Cell apoptosis was determined using flow cytometry (FCM) assay. The expression of lncRNA EBLN3P was upregulated while that of miR-655-3p was downregulated in human T-ALL cell lines and lncRNA EBLN3P negatively regulated miR-655-3p. LncRNA EBLN3P knockdown significantly inhibited proliferation, invasion, and migration of Jurkat cells and induced their apoptosis. Downregulating miR-655-3p reversed the effects of lncRNA EBLN3P knockdown on Jurkat cells. In conclusion, we confirmed for the first time that lncRNA EBLN3P is dysregulated in T-ALL cell lines, and lncRNA EBLN3P knockdown inhibited the malignant biological behaviors of T-ALL cells by up-regulating miR-655-3p.

## Graphical abstract

**Figure:**
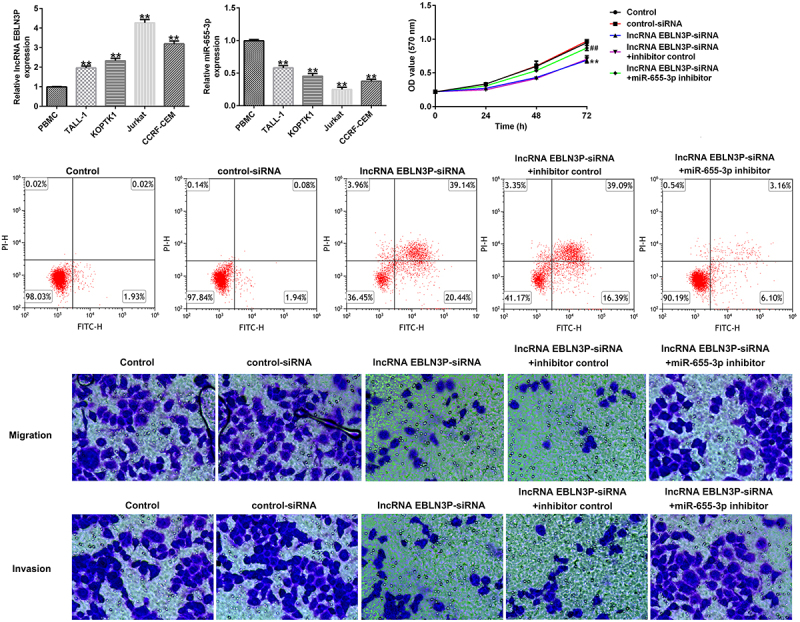

## Introduction

T-cell acute lymphoblastic leukemia (T-ALL) is a hematologic malignancy characterized by malignant hematopoietic cells that diffusely infiltrate the bone marrow and other tissues [1]. T-ALL accounts for 15% and 25% of all ALL cases in children and adults, respectively [2,3]. According to the different stages of T cell differentiation in the thymus, T-ALL can be divided into pro-T-, pre-T-, cortical-T-, and medullary-T-ALL [4]. Earlier reports indicated that mature T-ALL (cortical T-ALL) had a better prognosis than the immature T-ALL (pro/pre-T-ALL) [5,6]; however, modern chemotherapy and minimal residual disease (MRD)-based risk stratification have rendered the stage of development without prognostic significance. Early precursor T-cell acute lymphoblastic leukemia (ETP-ALL) was recognized as a new provisional entity of ALL by the World Health Organization (WHO) in 2016, with unique immunophenotypes and genetic characteristics [7]. Approximately 70% of pediatric and 50% of adult T-ALL patients achieve a long-term complete remission with intensive treatment regimens [^8–10^]. However, the prognosis for patients with relapsed T-ALL remains poor – one-third of patients suffer from recurrent disease, and the 5-year overall survival rate (OS) for adults is approximately 50%–55% [11,12]. Since narabine, approved in 2005, no new drugs have been specifically approved for relapsed T-ALL. The blast of proliferation and survival advantages of T-ALL cells arose from changes in signaling pathways [13].

Long noncoding RNAs (lncRNAs) are the largest subclass of noncoding RNAs ubiquitous in eukaryotic cells. LncRNAs are over 200 nucleotides in length and lack an open reading frame [14,15]. They regulate gene expression and participate in various cell signaling pathways by interacting with DNA, RNA, or proteins [16,17]. LncRNAs involved in cancer signaling pathways play important roles in tumor suppression or growth [18]. Transcriptome analysis has shown that lncRNAs exhibit highly specific spatiotemporal expression patterns of lineage across different tissues and cell types [19,20]. Increasing evidence has shown that lncRNAs are dysregulated in various cancers. Wang *et al*. revealed that 826 lncRNAs were abnormally expressed in bladder cancer, three of which were closely related to the OS of patients [21]. Liu *et al*. identified 145 lncRNAs to be abnormally expressed in esophageal cancer (ESCA), of which eight lncRNAs were associated with OS in ESCA patients [22]. Thus, lncRNAs could be used as reliable biomarkers and therapeutic targets to achieve more effective cancer treatments. The prominent types of lncRNAs have a common function – to regulate gene expression by sponging miRNAs [23,24]. LncRNA bornavirus-like nucleoprotein (lncRNA EBLN3P) has been reported to be abnormally expressed in various cancers. Dai *et al*. noted that lncRNA EBLN3P was overexpressed in osteosarcoma tissues and cell lines and promoted osteosarcoma progression by sponging miR-224-5p [25]. Li *et al*. revealed that lncRNA EBLN3P could promote the progression of liver cancer by competitively sponging miR-144-3p [26]. A study by Jiang *et al*. revealed that lncRNA EBLN3P promoted the recovery of damaged spiral ganglion neurons by competitively binding with miR-204-5p [27]. In breast cancer, the expression of lncRNA EBLN3P was downregulated, and its expression was related to better prognosis of OS and longer progression-free interval (PFI) [28]. Increasing evidence indicates that lncRNAs are associated with the pathogenesis and progression of T-ALL. The lncRNA CDKN2B-AS1 is upregulated in pediatric T-ALL peripheral blood mononuclear cells and promotes T-ALL development [29]. LncRNA NRIL was significantly upregulated in T-ALL samples, and its downregulation significantly inhibited the malignant biological behavior of T-ALL cells [30]. To date, the role of lncRNA EBLN3P in T-ALL has not been clarified.

LncRNAs can act as microRNA (miRNA) sponges, reducing their regulatory effect on mRNAs [31]. miRNAs are a class of noncoding RNAs (~22 nts) that could negatively regulate their target genes [32]. Bioinformatics analysis found that there are hundreds of miRNAs that bind to lncRNA EBLN3P, including miR-655-3p. Interestingly, miR-655-3p has been shown to participate in the occurrence and progression of many cancers, such as ovarian cancer, non-small cell lung cancer, and hepatocellular carcinoma [^33–35^], with its expression downregulated in all these cancers. The role of miR-655-3p in T-ALL has not been reported, but its expression is abnormally decreased in acute myeloid leukemia (AML) cells. MiR-655-3p silencing reversed the cytarabine (Ara-C)-induced proliferation inhibition and apoptosis promotion [36].

In the present study, we hypothesized that lncRNA EBLN3P plays a cancer-promoting role in T-ALL through regulating miR-655-3p expression. Therefore, in this study, we investigated the expression of lncRNA EBLN3P in human T-ALL cell lines and its role in the progression of T-ALL and its molecular mechanism.

## Materials and methods

### Clinical samples

22 cases clinical samples were obtained from Wuhan Fourth Hospital, Puai Hospital, Tongji Medical College, Huazhong University of Science and Technology from April 2020 to April 2021. 22 cases of blood samples were obtained from 22 T-ALL patients (age range, 4–28 years; 11 males, 11 females) and 22 healthy donors (age range, 3–32 years; 11 males, 11 females). The present study was approved by the Ethical Review Committee of Wuhan Fourth Hospital, Puai Hospital, Tongji Medical College, Huazhong University of Science and Technology. Written informed consents were obtained from all of the patients or legal guardians.

### Cell culture

The human T-ALL cell line (TALL-1, KOPTK1, Jurkat, CCRF-CEM) and human peripheral blood mononuclear cells (PBMCs) were purchased from the Department of Cell Biology, Institute of Basic Medical Sciences, Chinese Academy of Medical Sciences (Beijing, China). All cells were grown in RPMI-1640 medium (Thermo, cat no. 11,875,093) supplemented with 10% FBS (Fetal Bovine Serum; Thermo, cat no. 10,100,147), and incubated in a humidified atmosphere at 37°C and 5% CO_2_.

### Quantitative reverse transcription PCR (qRT-PCR)

Total RNA was extracted using Beyotime (cat no. R0011) reagent. We collected 5 × 10^6^ cells, resuspended and lysed them with 100 μl Beyozo reagent, added 20 μl chloroform, and incubated them for 3 min at room temperature. The supernatant obtained by centrifugation was mixed with 50 μl isopropanol and incubated for 10 min at room temperature. Finally, total RNA was precipitated using centrifugation and dissolved in RNase-free water, and its concentration was determined for later use. Quantitative reverse transcription PCR (qRT-PCR) was performed using the BeyoFast™ SYBR Green One-Step qRT-PCR Kit (cat no. D7286S). The reaction mixture was prepared according to the manufacturer’s instructions, and the experiment was performed using an Applied Biosystems 7500 Fast real-time PCR system. We used small nucleolar RNA U6 and GAPDH as internal controls for RNA and gene expression, respectively. Data for each sample was normalized to that of U6 or GAPDH. Relative quantification was performed using the 2^−ΔΔCt^ method [37]. Primer sequences were obtained from Sangon Biotech (Shanghai, China) and listed as following:

GAPDH forward, 5′-CATCATCCCTGCCTCTACTGG-3′; reverse, 5′-GTGGGTGTCGCTGTTGAAGTC-3′; U6 S, 5′-GGAACGATACAGAGAAGATTAGC-3′; Stem-loop-R, 5′-CTCAACTGGTGTCGTGGAGTC-3′; MMP-9 forward, 5′-TCCCCATCGCCATCCCC-3′; reverse, 5′-CACCATGGCCTCGGCTGG-3′; lncRNA EBLN3P forward, 5′-CAGACTAAAGGATCAAGCGAGA-3ʹ; reverse, 5′-ATCAATTGCCACAGGTTGAAGA-3ʹ; miR-655-3p forward, 5′-CAATCCTTACTCCAGCCAC-3′; reverse, 5′-GTGTCTTAAGGCTAGGCCTA-3′.

### Dual-luciferase reporter assay

The interaction between lncRNA EBLN3P and miR-655-3p was verified using a dual-luciferase reporter assay *in vitro* [38]. The wild type and mutant sequences of lncRNA EBLN3P were cloned downstream of the luciferase gene promoter (PGL3-lncRNA EBLN3P-wt or PGL3-lncRNA EBLN3P-mut). A dual-luciferase reporter assay was performed using HEK293T cells. Before transfection, HEK293T cells were inoculated into 12-well plates at 50% confluence and cultured for 16 h. Then, the cells were co-transfected with PGL3-lncRNA EBLN3P-wt, PGL3-lncRNA EBLN3P-mut, and miR-655-3p mimic or mimic control for 48 h. Luciferase activity was measured using a luciferase reporter assay kit (Abcam, cat no. ab228530).

### RNA pull-down assay

The interaction between lncRNA EBLN3P and miR-655-3p was verified using an RNA pull-down assay *in vitro* [39]. Jurkat cells were collected and lysed using sonication. The lncRNA EBLN3P probe was biotinylated with Pierce™ RNA 3ʹ-terminal biotinylation kit (Thermo, cat no. 20,160) and incubated with M-280 streptavidin magnetic beads (S3762, Sigma, USA) to generate probe-coated beads. The lncRNA EBLN3P probe-coated beads and oligo probe-coated beads were incubated with Jurkat cell lysis at 4°C overnight with the lncRNA EBLN3P anti-sense probe as a negative control. Finally, the bound RNA was eluted with a high-salt buffer solution, and miR-655-3p was quantified using RT-qPCR.

### Cell proliferation ability

Cell proliferation was determined using an MTT assay kit (Abcam, cat no. ab211091) [40]. The transfected cells were seeded into 96-well plates at a density of 5 × 10^3^ cells/well in 200 μl of culture medium and cultured for the indicated time (0, 24, 48, and 72 h). Then, the plates were centrifuged at 4°C at 1000 × g for 5 min in a microplate-compatible centrifuge, and the medium was replaced with a mixture of 50 μl serum-free media and 50 μl MTT reagent. The plates were cultured for 3 h and then centrifuged, and the MTT reagent-supplemented medium was removed. Finally, 150 μl MTT solvent was added to each well and incubated for 15 min at room temperature. Absorbance was measured at 570 nm wavelength.

### Western blot analysis

The expression of proteins in Jurkat cells was determined using Western blotting [41]. Cells were washed with pre-cold PBS buffer, lysed with M-PER reagent (Thermo, cat no. 78,501) containing phosphatase and protease inhibitor cocktail (Thermo, cat no. 78,440), and centrifuged at 12,000 rpm for 40 min at 4°C. The supernatant with a total protein content of 20 μg was separated on 12% SDS-PAGE. The protein was transferred to PVDF membranes using an XCell SureLock Mini-Cell electrophoresis system (Thermo) and blocked with 5% nonfat dry milk in TBS for 1 h at room temperature. The membranes were then incubated with primary antibodies (Abcam; 1:500 for Anti-cleaved caspase-3, cat. no. ab2302; 1:5000 for Anti-caspase-3, cat. no. ab32351; 1:5000 for Anti-MMP-9, cat. no. ab76003; 1:5000 for Anti-PCNA, cat. no. ab92552; 1:2500 for Anti-GAPDH, cat. no. ab9485) and secondary antibodies (Abcam; 1:5000, cat. no. ab97080). The enhanced chemiluminescence method (Cytiva) was used to detect immune response bands.

### Flow cytometry (FCM) analysis of cell apoptosis

Cell apoptosis was detected using the Annexin V-FITC/PI Apoptosis Detection Kit (GeneBio Systems, cat no. A211-01). In brief, 5 × 10^5^ transfected Jurkat cells were collected and washed with pre-cold PBS buffer. The cells were re-suspended in 100 μl 1X binding buffer, and 5 μl Annexin V-FITC and 5 μl PI staining solution were added. The mixture was incubated at room temperature for 10 min in the dark. Finally, 400 μl 1X binding buffer was added and the cells were analyzed by flow cytometry using FACSCalibur (BD Biosciences) within 1 h of staining [42].

### Transwell assay

The invasion and migration of Jurkat cells were determined by a transwell assay. In the cell invasion experiment, the processed Jurkat cells were collected and resuspended in a culture medium without FBS. Then, 200 μl cell suspension (containing 2 × 10^5^ cells) was plated on the top chamber of a transwell plate coated with Matrigel (Merck, cat. no. CLS3422) and 500 μl of the medium containing 10% FBS was added to the lower chamber. The cells were then cultured for 24 h. After incubation, the medium was gently removed, and the cells in the lower chamber were fixed with 4% paraformaldehyde for 30 min. The cells were then stained with 0.1% crystal violet for 20 min. Finally, the stained cells were observed and photographed using a confocal microscope. The procedure for the migration experiment was similar to that for the invasion experiment, except the plate was not coated with Matrigel [43].

### Statistical analysis

In this study, all experiments were independently repeated more than three times. Data are expressed as the mean ± standard deviation. Unpaired t-test and one-way ANOVA were used to analyze two groups and multiple groups of data, respectively, using SPSS 19.0 (SPSS Inc., Chicago, IL). A P value of <0.05 was considered statistically significant.

## Results

### LncRNA EBLN3P sponges miR-655-3p

**Figure 1.**
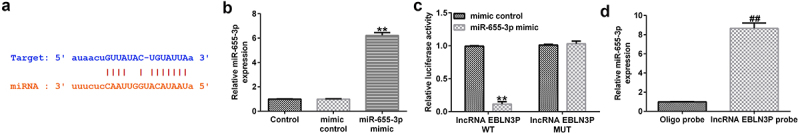
LncRNA EBLN3P sponges miR-655-3p.

The interaction between miRNAs, mRNAs, circRNAs, and lncRNAs reveals the complex mechanism of the occurrence and development of various cancers. As the expression of lncRNA EBLN3P and miR-655-3p was dysregulated in T-ALL patients, we hypothesized that there was an interaction between lncRNA EBLN3P and miR-655-3p in T-ALL cells. The functional binding site for miR-655-3p on lncRNA EBLN3P was predicted using the biological prediction website (http://starbase.sysu.edu.cn/index.php) (Figure 1(a)). The interaction was further verified using dual-luciferase reporter assays and RNA pull-down assays. We first confirmed that, compared with the mimic control group, miR-655-3p mimic significantly enhanced miR-655-3p levels in 293 T cells (Figure 1(b)). The dual-luciferase reporter assay showed that miR-655-3p mimic transfection resulted in a significant decrease in luciferase activity of the PGL3-lncRNA EBLN3P-wt vector, but cells with the PGL3-lncRNA EBLN3P-mut vector remained unaffected (Figure 1(c)). In the RNA pull-down assay, miR-655-3p was significantly enhanced in the lncRNA EBLN3P probe-coated group compared with the oligo probe-coated group (Figure 1(d)).

(A) The binding sites between lncRNA EBLN3P and miR-655-3p were predicted using StarBase (http://starbase.sysu.edu.cn/index.php). (B) The level of miR-655-3p in 293 T cells transfected with mimic control or miR-655-3p mimic was determined using qRT-PCR. (C) Dual-luciferase reporter assay was performed to verify the effects of miR-655-3p on the luciferase activity of PGL3-lncRNA EBLN3P-wt and PGL3-lncRNA EBLN3P-mut vectors. (D) RNA pull-down assay was used to determine the interaction between lncRNA EBLN3P and miR-655-3p *in vitro*. The results indicated that miR-655-3p could be pulled by beads coated with lncRNA EBLN3P probe but not the Oligo probe-coated beads. **p < 0.01 vs. Mimic control; ## p < 0.01 vs. Oligo probe.

### The expression of lncRNA EBLN3P and miR-655-3p in clinical samples and T-ALL cell lines

**Figure 2.**
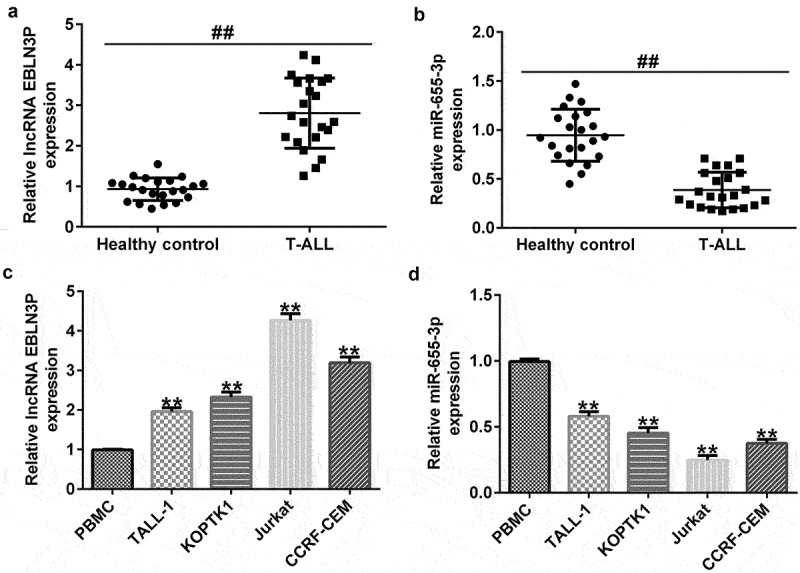
LncRNA EBLN3P was upregulated, while miR-655-3p was downregulated in clinical samples of T-ALL patients and T-ALL cell lines.

LncRNAs EBLN3P and miR-655-3p participate in the development of various cancers. Therefore, we investigated the expression of lncRNA EBLN3P and miR-655-3p in clinical samples from healthy donors and T-ALL patients, and in T-ALL cell lines. Compared with the healthy samples, the levels of lncRNA EBLN3P in the patient samples were significantly upregulated, while the level of miR-655-3p significantly down-regulated (Figure 2(a, b)). The results of the qRT-PCR assay indicated that the expression of lncRNA EBLN3P was significantly upregulated in human T-ALL cell lines (TALL-1-1.96 fold, KOPTK1-2.33 fold, Jurkat-4.26 fold, and CCRF-CEM-3.19 fold) compared with the human peripheral blood mononuclear cells (PBMCs) (Figure 2(c)). The expression of miR-655-3p was downregulated in human T-ALL cell lines (TALL-1-0.58 fold, KOPTK1-0.45 fold, Jurkat-0.25 fold, and CCRF-CEM-0.37 fold) compared with normal blood samples and PBMCs (Figure 2(d)), respectively. The human T-ALL Jurkat cell line with the most obvious expression changes of lncRNA EBLN3P and miR-655-3p was selected for further study.

The expression levels of lncRNA EBLN3P and miR-655-3p were determined using qRT-PCR. (A) The levels of lncRNA EBLN3P in the patient samples were significantly upregulated compared with the healthy samples. (B) The levels of miR-655-3p in the patient samples were significantly down-regulated compared with the healthy samples. (C) LncRNA EBLN3P was upregulated in T-ALL cell lines compared with the PBMCs. (D) MiR-655-3p was downregulated in T-ALL cell lines compared with PBMCs. ##p < 0.01; **p < 0.01 vs. PBMCs.

### miR-655-3p was negatively regulated by lncRNA EBLN3P in Jurkat cells

**Figure 3.**
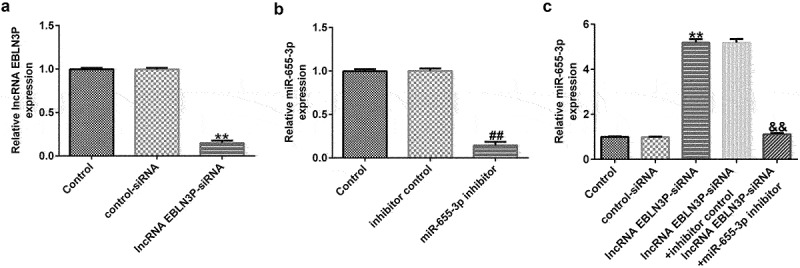
MiR-655-3p was negatively regulated by lncRNA EBLN3P in Jurkat cells.

To explore whether miR-655-3p was negatively regulated by lncRNA EBLN3P in Jurkat cells, Jurkat cells were transfected with control siRNA, lncRNA EBLN3P-siRNA, inhibitor control, miR-655-3p inhibitor, lncRNA EBLN3P-siRNA + inhibitor control, and lncRNA EBLN3P-siRNA + miR-655-3p inhibitor for 48 h. qRT-PCR was used to determine transfection efficiency. Compared with control siRNA, lncRNA EBLN3P-siRNA transfection significantly diminished lncRNA EBLN3P expression in Jurkat cells (Figure 3(a)). The expression of miR-655-3p was inhibited by the miR-655-3p inhibitor in Jurkat cells compared with the inhibitor control (Figure 3(b)). In lncRNA EBLN3P-siRNA transfected Jurkat cells, the expression of miR-655-3p was markedly increased, and this increase was reversed by the miR-655-3p inhibitor (Figure 3(c)).

Jurkat cells were transfected with control siRNA, lncRNA EBLN3P-siRNA, inhibitor control, miR-655-3p inhibitor, lncRNA EBLN3P-siRNA + inhibitor control, and lncRNA EBLN3P-siRNA + miR-655-3p inhibitor for 48 h, and the transfection efficiency was determined using qRT-PCR. (A) LncRNA EBLN3P-siRNA transfection significantly decreased the expression of lncRNA EBLN3P in Jurkat cells compared with the control siRNA transfection. (B) Transfection with miR-655-3p inhibitor significantly decreased the expression of miR-655-3p in Jurkat cells compared with the cells transfected with the inhibitor control. (C) The expression of miR-655-3p was upregulated after transfection with lncRNA EBLN3P-siRNA, and the enhancement was reversed by co-transfection with miR-655-3p inhibitor. **p < 0.01 vs. Control-siRNA; ## p < 0.01 vs. inhibitor control; &&p < 0.01 vs. lncRNA EBLN3P-siRNA + inhibitor control.

### LncRNA EBLN3P-siRNA inhibits proliferation and induces apoptosis of Jurkat cells by upregulating miR-655-3p

**Figure 4.**
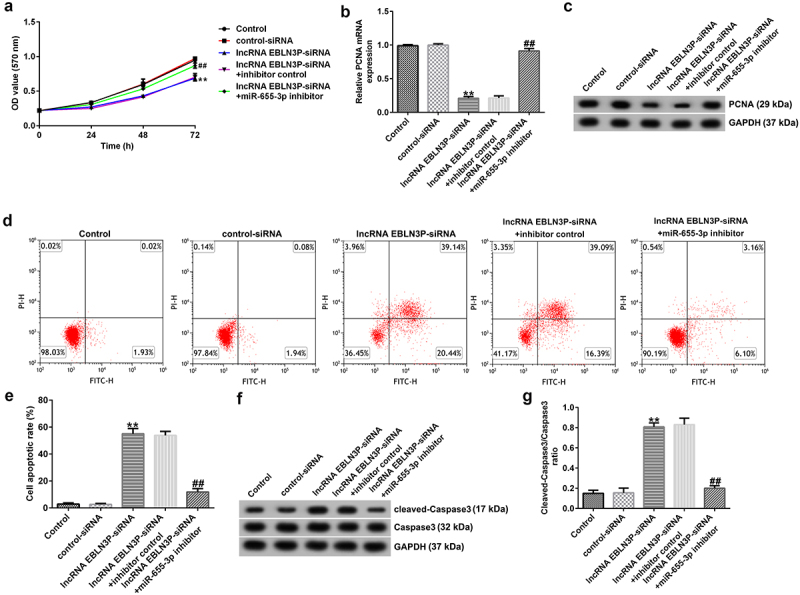
LncRNA EBLN3P knockdown inhibits the proliferation and induces apoptosis of Jurkat cells by upregulating miR-655-3p.

To investigate the role of lncRNA EBLN3P knockdown on the proliferation and apoptosis of Jurkat cells, Jurkat cells were transfected with control-siRNA, lncRNA EBLN3P-siRNA, lncRNA EBLN3P-siRNA + inhibitor control, and lncRNA EBLN3P-siRNA + miR-655-3p inhibitor for 48 h. Cell proliferation ability was determined using the MTT assay at the indicated time points (0, 24, 48, and 72 h). The results revealed that the proliferation ability of Jurkat cells was significantly inhibited by lncRNA EBLN3P-siRNA transfection compared with control-siRNA transfection, while the inhibitory effect of lncRNA EBLN3P-siRNA transfection could be reversed by miR-655-3p inhibitor transfection (Figure 4(a)). The mRNA and protein expression of proliferating cell nuclear antigen (PCNA) were determined using qRT-PCR and Western blot assays, respectively. We observed a decreased mRNA and protein expression of PCNA in Jurkat cells after transfection with lncRNA EBLN3P-siRNA and lncRNA EBLN3P-siRNA co-transfected with miR-655-3p inhibitor rescued the mRNA and protein expression of PCNA (Figure 4(b, c)). Apoptosis of Jurkat cells was detected using flow cytometry (FCM) analysis. LncRNA EBLN3P-siRNA transfection significantly induced apoptosis of Jurkat cells compared to control siRNA, while co-transfection with miR-655-3p inhibitor inhibited the apoptosis induced by lncRNA EBLN3P-siRNA (Figure 4(d, e)). The expression of cleaved-caspase3 and caspase3 was determined using Western blotting. LncRNA EBLN3P-siRNA transfection obviously increased the protein expression of cleaved-caspase3 (figure 4(f)) and the ratio of cleaved-caspase3/caspase3 (Figure 4(g)) in Jurkat cells compared to control-siRNA transfection, and this increase was reversed by co-transfection with miR-655-3p inhibitor.

Jurkat cells were transfected with control-siRNA, lncRNA EBLN3P-siRNA, lncRNA EBLN3P-siRNA + inhibitor control, and lncRNA EBLN3P-siRNA+miR-655-3p inhibitor for 48 h. (A) MTT assay was used to detect the cell proliferation ability. (B) and (C) The mRNA and protein levels of PCNA were detected using qRT-PCR and Western blotting, respectively. (D) and (E) FCM assay was used to determine cell apoptosis. (F) Western blot assay was used to measure the protein expression of cleaved-caspase3 and caspase3. (G) The ratio of cleaved-caspase3/caspase3. **p < 0.01 vs. Control-siRNA; ## p < 0.01 vs. lncRNA EBLN3P-siRNA + inhibitor control.

### LncRNA EBLN3P-siRNA inhibits invasion and migration of Jurkat cells by upregulating miR-655-3p

**Figure 5.**
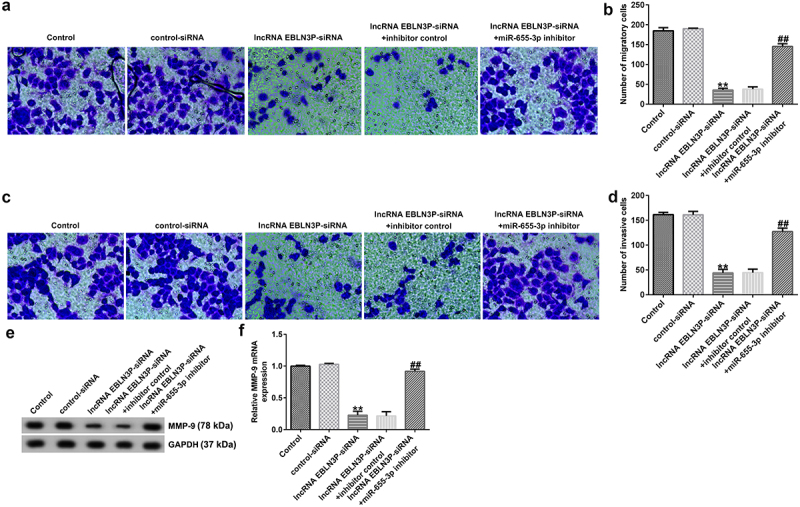
LncRNA EBLN3P knockdown inhibits invasion and migration of Jurkat cells by upregulating miR-655-3p.

To study the role of lncRNA EBLN3P knockdown on the invasion and migration of Jurkat cells, Jurkat cells were transfected with control-siRNA, lncRNA EBLN3P-siRNA, lncRNA EBLN3P-siRNA + inhibitor control, and lncRNA EBLN3P-siRNA + miR-655-3p inhibitor for 48 h. The invasion and migration of Jurkat cells were determined using a transwell assay. The results indicated that the migration (Figure 5(a, b)) and invasion (Figure 5(c, d)) of Jurkat cells were inhibited by lncRNA EBLN3P-siRNA transfection compared with the control siRNA group, and the inhibitory effect of lncRNA EBLN3P-siRNA on cell invasion and migration could be reversed by co-transfection with miR-655-3p inhibitor. Matrix metalloproteinases (MMPs) are a family of zinc-dependent proteases, and MMP-9 is one of the main members of the family [44]. Activated MMP-9 can degrade basement membrane (BM) type IV collagen to promote the invasion and migration of tumor cells [45]. The protein and mRNA expression of MMP-9 in Jurkat cells were detected using Western blotting and qRT-PCR, respectively. LncRNA EBLN3P-siRNA transfection significantly inhibited the expression and transcription of MMP-9 (Figure 5(e, f)), while co-transfection with miR-655-3p inhibitor abolished the inhibitory effects on MMP-9.

Jurkat cells were transfected with control-siRNA, lncRNA EBLN3P-siRNA, lncRNA EBLN3P-siRNA + inhibitor control, and lncRNA EBLN3P-siRNA + miR-655-3p inhibitor for 48 h. (A) and (B) Cell migration and (C) and (D) invasion were determined using a transwell assay. (E) and (F) Protein and mRNA expression of MMP-9 was detected using Western blot assay and qRT-PCR analysis. **p < 0.01 vs. Control-siRNA; ## p < 0.01 vs. lncRNA EBLN3P-siRNA + inhibitor control.

## Discussion

Newly diagnosed T-ALL patients show improvement as the treatment progresses, including glucocorticoids, asparaginase, and central nervous system targeted therapy. However, the prognosis of patients with relapsed T-ALL remains poor [11,12,46]. Therefore, further study on the pathogenesis of T-ALL will be helpful in the early identification of the disease, discovery of new therapeutic targets, and development of more efficacious drugs to improve the prognosis of the patients.

Dysregulated lncRNAs involved in different regulatory mechanisms may serve as new biomarkers for distinguishing between ALL subgroups and determining the prognosis [47,48]. The lncRNA EBLN3P is dysregulated in many cancers, including osteosarcoma, liver, and breast cancers [25,26,28]. However, the role of EBLN3P in T-ALL remains unclear.

MiR-655-3p has been confirmed to be involved in the occurrence and development of various tumors [^33–35^]. For example, miR-655-3p inhibited proliferation and migration of ovarian cancer cells by targeting RAB1A [33]. miR-655-3p inhibits non-small cell lung cancer cell migration and invasion by targeting pituitary tumor-transforming 1 [34]. Besides, miR-655-3p plays a tumor suppressor role in acute myeloid leukemia cells [36]. These results suggest that miR-655-3p may inhibit the proliferation, migration and invasion of T-ALL cells. As a prominent function of lncRNAs is to regulate gene expression by sponging miRNAs [23,24,49], we investigated the correlation between lncRNA EBLN3P and miR-655-3p in T-ALL cells. StarBase (http://starbase.sysu.edu.cn/index.php) predicted an interaction between lncRNA EBLN3P and miR-655-3p that was further confirmed by luciferase reporter assays and RNA pull-down assays. In this study, we found that the expression of lncRNA EBLN3P is upregulated in clinical samples of T-ALL patients and in human T-ALL cell lines, while miR-655-3p is downregulated in clinical samples of T-ALL patients and in human T-ALL cell lines.

We then conducted several experiments to explore the effects of miR-655-3p and lncRNA EBLN3P on the malignant biological behaviors of T-ALL cells. Dysregulated cell proliferation, together with inhibition of apoptosis, provides the minimal ‘platform’ necessary to support further tumor progression [50]. Migration and invasion of cancer cells into surrounding tissue and vasculature is an important initial step in cancer metastasis, and metastasis is the leading cause of cancer related death [51]. In this study, the data indicated that EBLN3P knockdown significantly inhibited malignant biological behaviors (proliferation, invasion, and migration) and induced apoptosis of T-ALL cells, and all the effects induced by lncRNA EBLN3P knockdown could be reversed by downregulating miR-655-3p. Moreover, cell proliferation and apoptosis related markers PCNA and Caspase3, and metastasis related gene MMP-9 were determined. PCNA is an essential protein involved in multiple processes of DNA metabolism, and targeting PCNA has been shown to be an effective strategy to inhibit tumor cell proliferation [52]. Caspases are key mediators of apoptosis, and among them, caspase-3 is a frequently activated death protease that catalyzes the specific cleavage of many key cellular proteins [53]. MMP-9 is a protease with a key role in tumor progression and metastasis [54]. Our findings indicated that lncRNA EBLN3P knockdown significantly decreased PCNA and MMP-9 expression, while enhanced cleaved-Caspase3 expression in T-ALL cells, and all these changes were significantly revised by miR-655-3p inhibitor.

However, we did not explore the more in-depth molecular mechanisms of the role of miR-655-3p in T-ALL cells (targets of miR-655-3p were not analyzed), and this was a limitation of the present study. We will perform this issue in the future.

## Conclusion

Our study is the first to demonstrate that lncRNA EBLN3P is dysregulated in T-ALL, and lncRNA EBLN3P knockdown may inhibit the malignant biological behaviors of T-ALL cells by preventing the sponging of miR-655-3p, providing a new potential therapeutic target for T-ALL.

## Data Availability

All datasets used and/or generated during the current study are available from the corresponding author on reasonable request.
